# Molecular mechanisms of system responses to novel stimuli are predictable from public data

**DOI:** 10.1093/nar/gkt938

**Published:** 2013-10-31

**Authors:** Samuel A. Danziger, Alexander V. Ratushny, Jennifer J. Smith, Ramsey A. Saleem, Yakun Wan, Christina E. Arens, Abraham M. Armstrong, Katherine Sitko, Wei-Ming Chen, Jung-Hsien Chiang, David J. Reiss, Nitin S. Baliga, John D. Aitchison

**Affiliations:** ^1^Seattle Biomedical Research Institute, Seattle, WA 98109-5219 USA, ^2^Institute for Systems Biology, Seattle, WA 98109-5240 USA, ^3^The Key Laboratory of Developmental Genes and Human Disease, Ministry of Education, Institute of Life Science, Southeast University, Nanjing 210096, China and ^4^Department of Computer Science and Information Engineering, National Cheng Kung University, Tainan 704, Taiwan

## Abstract

Systems scale models provide the foundation for an effective iterative cycle between hypothesis generation, experiment and model refinement. Such models also enable predictions facilitating the understanding of biological complexity and the control of biological systems. Here, we demonstrate the reconstruction of a globally predictive gene regulatory model from public data: a model that can drive rational experiment design and reveal new regulatory mechanisms underlying responses to novel environments. Specifically, using ∼1500 publically available genome-wide transcriptome data sets from *Saccharomyces cerevisiae*, we have reconstructed an environment and gene regulatory influence network that accurately predicts regulatory mechanisms and gene expression changes on exposure of cells to completely novel environments. Focusing on transcriptional networks that induce peroxisomes biogenesis, the model-guided experiments allow us to expand a core regulatory network to include novel transcriptional influences and linkage across signaling and transcription. Thus, the approach and model provides a multi-scalar picture of gene dynamics and are powerful resources for exploiting extant data to rationally guide experimentation. The techniques outlined here are generally applicable to any biological system, which is especially important when experimental systems are challenging and samples are difficult and expensive to obtain—a common problem in laboratory animal and human studies.

## INTRODUCTION

Systems biology promises to impact all areas of biological sciences, including ecology (1), biotechnology (2) and medicine (3,4). Such studies are generally characterized by large-scale data generation followed by analysis, modeling and prediction. Typically, data are deposited in publically accessible databases, which present an opportunity to use data integration strategies to more fully exploit the rich data sets generated by different laboratories. However, large ‘omics data sets are often underused because the conditions under which the data were originally generated may not be considered directly relevant to a new question or condition under study. Thus, it remains a significant challenge to effectively use such data sets to generate genome-scale predictive models that are relevant to novel conditions. Ideally extant data can be analysed to illuminate comprehensible molecular networks that suggest specific experiments and reveal novel mechanistic behavior (5). In this study, we provide a template for molecular systems approaches to exploit large public data sets, which are increasingly available for numerous and varied biological systems. Such approaches are critically important to further research into human health. Applications include fundamental research into genetic regulation, identification of drug targets in diseased or pathogen-infected cells and engineering microorganisms for remediation or production of biomaterials.

Gene regulatory influence network (GRIN) models are typically constructed by integrating high-throughput experiments with existing biological knowledge to fuel a virtuous cycle where the GRIN informs biological experimentation, which yields yet more informative models for new experiments (6). Large databases of high-throughput experiments abound; as of Spring 2013, the Gene Expression Ominibus [GEO (7)] contained >920 000 expression samples. Databases like these can inform an initial GRIN, but the specific interactions are highly dependent on the environment (8), thus it is necessary to construct models that include environmental influences—so called environment and gene regulatory influence networks (EGRIN).

Here we demonstrate that a yeast EGRIN can be generated from a large publically available mRNA data set, and that this EGRIN can make accurate predictions of gene expression under novel experimental conditions. We use this network to identify factors that regulate a process of interest. We then show that a small number of additional condition-specific experiments can refine these predictions and result in an ever more accurate model. The factors selected by this refined model were then examined experimentally by gene disruption and genome-wide binding (ChIP-chip) studies further improving the mechanistic basis and predictive power of the model. Using this process, free publically available data and relatively cheap gene expression experiments identify relevant regulators to explore with more detailed (and expensive) experiments. This approach stands in contrast with others that integrate different data types (generally generated under the same environmental conditions) to create the initial model (9,10), and thus miss the full power presented by consortium data sets.

**Figure 1.**
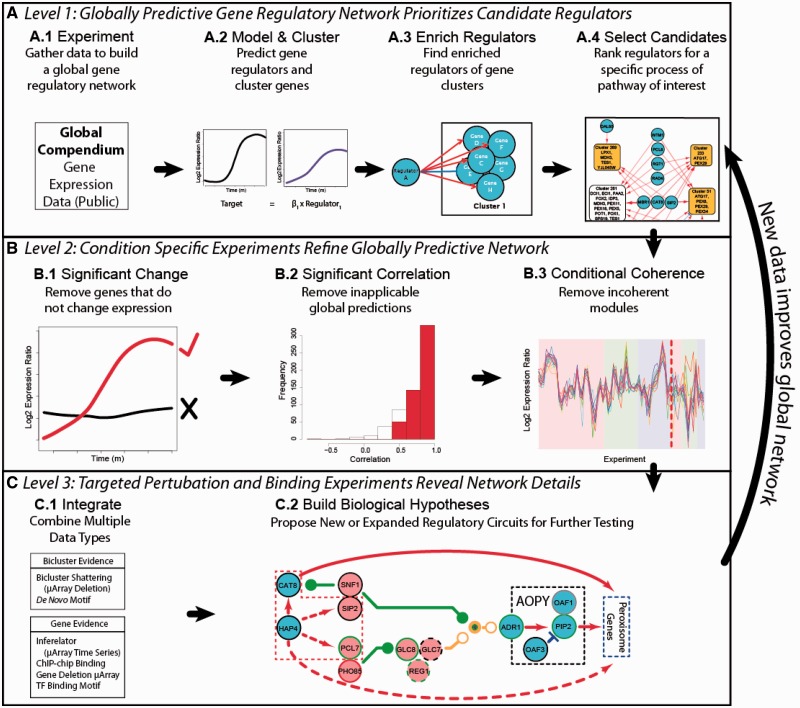
EGRIN overview and application. The three levels of EGRIN. (**A**) Level 1: (A.1) mRNA experiments are used to (A.2) construct a globally predictive network using cMonkey and Inferelator. (A.3) Regulators are chosen that are statistically overrepresented as regulating genes in the clusters. (A.4) A ranked list of candidates for further experimentation is generated from regulators of interesting clusters. (**B**) Level 2: (B.1) The initial data set is filtered to only include genes that change significantly during condition-specific experiments. (B.2) The predicted regulation generated by the linear regression is filtered to only include targets that are well predicted during the condition-specific experiments. (B.3) Scores for candidates to be considered for further experimentation are weighted by the coherence of clusters during condition-specific experiments. (**C**) Level 3: (C.1) Experimental results are combined with other available data to construct (C.2) a gene-level regulatory network. Once the experiments for (B) and (**C**) are completed, the newly discovered biology is fed back into (A) to improve predictions for additional conditions.

Our method is based on a previous study that constructed EGRINs for *Halobacterium salinarum* (11). The basic strategy for EGRIN generation is 2-fold. First conditionally coherently expressed subsets of genes (i.e. biclusters) are identified as forming putatively coregulated modules that are coherent across some environmental conditions. These modules can be often associated with specific aspects of cellular function through enrichment of gene ontology (GO) terms for composite genes (12). Second, direct gene regulatory influences by transcription factors (TFs) are inferred based on changes in mRNA expression data for TFs within each module (11). Our strategy follows this approach, but, because of the prevalence of posttranslational control mechanisms in eukaryotic systems, it also considers other forms of regulation (such as kinases and other posttranslational modifiers) that may influence mRNA expression (13–15). From this list of regulators, which implies a large number of possible combinations, the EGRIN was used to select a manageable number for detailed experimentation. It was then augmented with additional data types to build a more detailed model of gene regulation through an iterative three-level strategy (presented in Figure 1); and thus turn low-resolution global data into condition-specific predictions.

Importantly, as *Saccharomyces cerevisiae* is a common model system for molecular cell biology and genetics that is exploited in synthetic biology, the global yeast EGRIN has wide applicability. We demonstrate its utility by generating insight into peroxisome biogenesis and function. Peroxisome biogenesis is a tightly regulated and highly integrated process in numerous cell types from yeasts to humans, and regulated peroxisome biogenesis is fundamentally important to human health. Peroxisomes perform many essential and diverse functions in eukaryotic cells, the most notable of which is the β-oxidation of fatty acids. Importantly, they are dynamic—proliferating in response to different environmental cues, including fatty acid exposure in yeasts (16–20). Thus peroxisomes are essential for normal human development and peroxisomal defects lead to severe neuropathologies (21). The varied roles that peroxisomes play in different aspects of cell biology and cellular function continue to be uncovered (21–24). Therefore, applying the yeast EGRIN to study peroxisomes is useful for understanding human health and disease.

Here, we demonstrate (i) a yeast EGRIN that accurately predicts gene expression across a broad array of novel environmental conditions (i.e conditions not probed as part of the data set used to construct the model) and identifies factors that regulate peroxisome-annotated genes; (ii) filters based on condition-specific experiments that refine the EGRIN and make it more accurate; (iii) five novel regulators of peroxisomes identified by the EGRIN and confirmed by gene deletion studies; (iv) novel aspects of peroxisome regulation; and (v) novel hypotheses regarding specific mechanisms responsible for mediating condition-specific cellular responses.

The resulting gene regulatory networks and raw data are available online as well as the R scripts used in this analysis (http://AitchisonLab.com/YeastEGRIN). Thus, we make public our approach to establish a large-scale predicted regulatory network from public data. This network is sufficiently predictive to suggest useful experiments for elucidating molecular mechanisms that confer specific phenotypes under novel environmental conditions. The experimental results are then fed back into the large-scale network to improve the overall predictive power.

## MATERIALS AND METHODS

This article combines both computational and biological methods. The computational methods used in this research, cMonkey and Inferelator (25,26), were originally developed to study *H. salinarum* (11). We adapted these tools to eukaryotic *S. cerevisiae* and included a number of changes detailed below. Unless otherwise noted, all algorithms developed for this research were implemented in the R programming language (27). All *P*-values were subject to standard false discovery rate controls as appropriate. Plots were generated using R (27), regulatory diagrams generated using Cytoscape (28) and all images prepared using Adobe Illustrator CS5. The R scripts used for this research contain additional details not presented in this text.

### Biclustering

cMonkey biclusters are based on multiple mRNA expression experiments, such as from microarrays (25). The genes are clustered into putatively coregulated units based on mRNA expression coherence, known network interactions and common promoter regions TF binding motifs. By default, the network interactions include protein–protein interactions from the STRING database. As ChIP-chip data became available, we supplemented the network interactions by indicating which genes were bound by the same TF under the same conditions. Two sets of cMonkey biclusters are available, one built on the 1516 compendium-based experiments and one built on 70 condition-specific experiments.

The condition-specific biclusters consist of 30 yeast-in-glucose experiments that simulated the Low Sugar (LS) condition, 19 Early Oleate (EO), and 21 Late Oleate (LO) yeast-in-oleate experiments. These condition-specific experiments came from three sources: 17 previously published chemostat experiments (29), 8 previously published batch culture experiments (18) and 15 novel batch culture experiments. None of these experiments were in the 1516 experiments in the compendium and none of the conditions tested in these experiments is known to cause peroxisome proliferation.

For each experiment, we constructed background distributions of the expected variance for 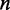 genes, where 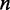 is the number of genes in a given cluster. By comparing the variance for each experiment in a bicluster to the background distribution, we determined which experiments were significantly coherent in that cluster (*P*-value cutoff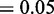). If more than half of the experiments for a condition (LS, EO or LO) were included in a cluster, we considered that cluster coherent under that condition.

### Inferred regulation

Inferelator (26,30,31) is a linear regression program that uses the mRNA expression levels of TFs or other regulators to predict the expression level of a target gene (or mean expression level of a bicluster). For this research, we replaced the linear regression algorithm with elastic net (32) regression setting 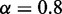 using the *lars* package (33). The elastic net is preferable to the old LASSO method because it does not select a predefined number of parameters and does not tend to select one of a number of high correlated regulators. Due to difficulties arising from mixing chemostat with batch culture experiments and insufficient temporal resolution in the experiments we set the decay constant (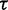) to zero. To limit the resulting increased number of predictions, we turned off the ‘and’ logic embedded in Inferelator. Microarrays are subject to noise, so for best results on gene-level predictions, our version of Inferelator predicts only those regulators or targets whose mRNA levels change significantly during the course of the experiment. We calculate significance using the lambda (34) when available or an estimated lambda fit to a chi-squared distribution (degrees of freedom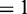, 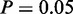) when no lambda is available. Gene expression data are normalized using z-score normalization (a form of quantile normalization that ensures that all distributions have the same mean and standard deviation) before being analyzed with cMonkey or Inferelator. Gene expression profiles are prepared for visualization by smoothing them with the loess algorithm (35).

### Evaluating inferred regulation

Inferelator provides a linear equation with coefficients that show which factors promote or repress a target. With the setting described above, the Inferelator equation may be presented as follows:

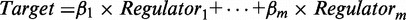

where 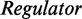 is one of 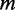 putative regulators of a 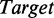 gene or bicluster. After regression and shrinkage, those 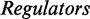 with non-zero coefficients (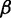
s) are considered predicted regulators of 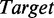. 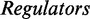 with positive 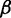
s are considered activators and those with negative 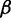
s are considered repressors.

We use four metrics to evaluate the accuracy of these linear equations in light of microarray deletion experiments where a 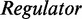 has been deleted. 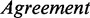 refers to the fraction of times that 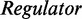 was predicted to be a activator or repressor of a 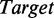 gene and the expression level of 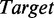 was decreased or increased (respectively) when 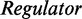 was deleted. Formally, 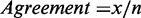 where 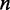 is the number of target genes where 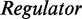 has a nonzero coefficient in the linear equation and 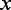 is the number of times that the coefficient and the log expression ratio of the target gene have different signs. 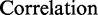 is the Pearson correlation coefficient between the target expression ratio and −1 times the coefficient for the 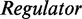. 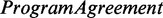 measures the accuracy of the entire linear equation for each target in which 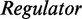 has a nonzero coefficient. The target gene expression is predicted by the linear equation based on the expression levels of all putative regulators in the deletion experiment, including 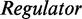. Thus 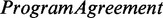 refers to the fraction of times that a target gene was correctly predicted to be up- or down-regulated in a deletion experiment. 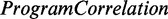 is the Pearson’s correlation coefficient between the predicted and actual expression levels of all genes for which 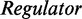 has a nonzero coefficient. These metrics are most prominently displayed in the Supplementary Data.

### Evaluation of clustering efficacy

Table 3 was generated by comparing the accuracy of cluster-level predictions with gene-level predictions. If Inferelator predicted that a TF regulated a gene, and that gene changed significantly in expression when that TF was deleted, then we considered that a true positive (TP). Similarly, all other receiver operator characteristics (ROCs) were generated for these gene level predictions. We then evaluated the predictions at a cluster level: if a bicluster was significantly enriched (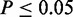) for genes predicted by Inferelator to be regulated by a TF, and a significant number of those genes significantly changed in expression when that TF was deleted, then we considered that a TP. Similarly, all other ROCs were generated for these cluster-level predictions. *P*-values were calculated using a Wilcoxon signed-rank test.

### Combining predictions

Inferelator predictions made based on different data sets may be combined by combining the terms in the linear equations so long as those predictions share common 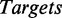. For purposes of this study, coefficients from the condition-specific data were given priority. For example, if Inferelator produces

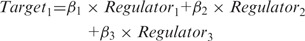

from the compendium data set and

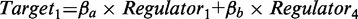

from the condition-specific data set, then the combined regulatory program would be

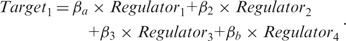

Note that 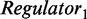 appears in both equations, but only 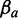 (the condition-specific coefficient) appears in the combined regulatory program. If 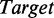 is a gene, then combined predictions may then be assigned to biclusters by comparing the number of 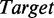 genes for a given 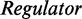 in a bicluster to a background distribution.

At Level 2, we combined our gene-level predictions using this method. We calculated a *P*-value by comparing the number of genes in each bicluster predicted to be regulated by a TF to a distribution of the number of genes that would be expected to be predicted to be regulated by that TF if the genes in the bicluster were selected at random. If a TF-bicluster pair had a 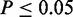, then that TF was considered to be a predicted regulator of that bicluster.

### Predicted regulators of peroxisomes

We ranked the predicted regulators of peroxisomes with a scoring function that combined the *P*-value of a predicted bicluster regulator with the conditional coherence for those biclusters enriched with peroxisomal genes.



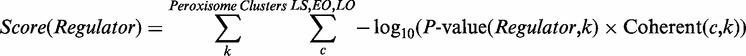

Where 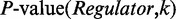 is the *P*-value calculated by comparing the Inferelator predicted regulation of the gene cluster 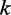 by 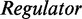 to the background distribution of genes regulated by 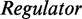. 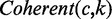 is 1 if cluster 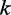 is coherent (*P*-value cutoff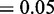) under condition 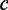 or 0 otherwise. These scores are shown for all regulators in Table 2.

### Streams of evidence

High-throughput and computational derived biological experiments tend to produce noisy results. To arrive at strong evidence for regulation, it is helpful to consider regulatory relationships that are reinforced by multiple streams of evidence (i.e. experimental types). This is particularly true when one stream of evidence shows direct regulation, such as a ChIP-chip experiment, and another stream of evidence shows indirect regulation, such as a microarray deletion experiment. We considered three streams of indirect evidence: one based on Inferelator predictions and two based on microarray deletion experiments. We also considered three streams of direct evidence: one based on ChIP-chip experiments and two based on TF Binding Motif presence. Since multiple genes may share the same promoter region, it is particularly important to include another stream of evidence when considering direct evidence.

### Evidence of indirect regulation: wild-type microarray time series

The 15 novel batch-culture experiments described in the Biclustering section were prepared as described in Strains and growth conditions but harvested at times other than just LS, EO, LO. Specifically, they were harvested at 0.5, 1, 1.5, 2, 2.5, 2.5, 2.75, 2.75, 3, 3, 3, 3, 3.5, 4 and 5 h after oleate induction (Supplementary Table S3). mRNA was purified using ethanol precipitation with a Qiagen Mini-prep kit. cDNA was labeled using Invitrogen Alexa 555 and 647 and bound to Agilent two color arrays. All experiments used dye-flipping, so each time point was compared with a LS reference point twice. The resulting microarray expression ratios were analyzed with Inferelator and cMonkey as described earlier in Materials and Methods.

### Evidence of indirect regulation: deletions microarrays

Yeast TF deletion strain cultures were prepared as described in Strains and growth conditions and analysed by microarrays as described in Evidence of indirect regulation: wild-type microarray time series. Two biological and two technical replicates without dye-flipping were performed for the *cat8*, *hap4* and *sps18* experiments, other experiments shown (excluding those previously published) each had only a single dye-flipped replicate. The *cat8*, *hap4* and *sps18* deletion experiments were performed before the EGRIN was constructed but never published. They were measured against a 0-h (LS) control, so it was necessary to convert the expression when they were to be compared against 0.5-h (EO) or 5-h (LO) controls. To do this, we subtracted the log expression ratios of wild-type EO and LO over LS controls from the deletion expressions. These data are presented in Supplementary Table S4.

We fit the resulting deletion expression values to a normal distribution and assigned *P*-values using both tails of the distribution. Statistically significant regulation for 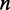 genes was calculated based on a hypergeometric distribution with a *P*-value cutoff of 0.05 using false discovery rate multiple-testing controls.

An additional stream of regulatory evidence was calculated using bicluster shattering. Shattering refers to the deletion of a TF disrupting the mRNA expression coherence of a bicluster. To detect this, we built a distribution of gene expression variances in each cluster for the LS, EO and LO conditions described previously. If the variance for the genes in the LS, EO or LO deletion experiments was >95% of the variances for that cluster, then the deleted TF was said to regulate the bicluster based on bicluster shattering. Thus, if genes were particularly tightly coregulated in a given cluster, it would be relatively easy for a deletion to shatter that cluster, but if the genes were not tightly coregulated, then it would be difficult for a deletion to shatter that cluster. If a gene is in multiple biclusters, the lowest *P*-value is used. These results are presented in Supplementary Table S5.

### Evidence of direct regulation: ChIP-chip binding

ChIP-chip experiments were performed using a modified version of a ChIP-chip protocol previously described (18). Cells with myc-tagged regulators were harvested as described in Strains and Growth Conditions and fixed in a 1% formaldehyde solution. Fixed cells were lysed with glass beads and the DNA sheared with sonication. Tagged fragments were collected by Invitrogen Dynabeads and the cross-linking reversed by heat. Immunopurified samples and whole-cell extracts were labeled using the Kreatech ULS aRNA Fluorescent Labeling DNA Kit as per the standard protocols. Microarrays were processed in-house and interpreted using the MeDiChI (36) algorithm to assign *P*-values to binding events. These results are presented in Supplementary Table S6.

Regulation was assigned if a binding event occurred in the region between 1000 bp upstream of a gene and 100 bp after the transcription start site. Those binding events assigned a 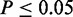 by MeDiChi were considered strong evidence and those with any other *P*-value were considered weak evidence. When there were multiple hits for a single TF-target pair, the lowest *P*-value was selected. To determine if a TF regulated a bicluster containing 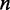 genes, a score was calculated for each gene in the cluster by taking the negative log of the *P*-value. A background distribution of expected scores was calculated for 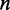 genes selected at random and regulation was assigned if the score was in the top 5% of expected scores.

### Evidence of direct regulation: TF binding motif

We downloaded known yeast TF binding motifs probability weight matrices from the YEASTRACT database (37–39) and supplemented them with motifs appearing in Saccharomyces Genome Database (SGD) (40). We searched the promoter regions for all known yeast genes using the Bioconductor Biostrings package (41). Biostrings provided functionality to score sequences in the promoter region (1000 bp upstream of a gene and 100 bp after the transcription start site) against existing probability weight matrices describing known binding motifs using a sliding window. These scores were compared with a background model of expected scores to calculate a *P*-value. Each of the resulting matches with a 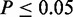 (Bonferroni corrected) was considered a hit. Some genes would thus have multiple hits from a given TF, corresponding to multiple possible binding sites for that TF in the promoter region. As the existence of multiple binding sites has been previously observed as a method of regulation in yeast (42), theoretically models of TF regulation that include multiple bindings are more accurate (43,44), and multiple hits increased the probability that a gene’s expression will significantly change when the TF is deleted (data not shown); we calculated a final *P*-value for each TF with target gene as follows: 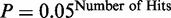. A resulting 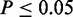 (again subject to Bonferroni correction) was considered evidence for regulation. Additionally, we recalculated the background distributions and used *P*-value of the best hit for each TF-gene pair, but with a *P*-value cutoff of 0.01. We considered a hit using either method evidence for the presence of a binding motif. These results are presented in Supplementary Table S7.

An additional stream of regulatory evidence was determined based on the *de novo* motifs detected by cMonkey (25). Those detected motifs with an e-value 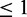 were run against the ‘macisaac’ and ‘scpd’ yeast motif databases using TomTom (45). The resulting *P*-values are subjected to a cutoff value of 0.05 to determine regulation. If a TF regulates a cluster, it is considered to regulate all genes in that cluster.

### Strains and growth conditions

The biological methods used in this research consisted of ChIP-chip and microarray experiments and were conducted using batch culture oleate induction (18). All yeast deletion strains were taken from the MATalpha yeast deletion library (46) and verified by polymerase chain reaction. BY4742 (47) was used for all wild-type strains. Cells for the ChIP-chip and microarray experiments were grown overnight in Yeast Extract Peptone Dextrose (YPD) solution, and transferred to S. cerevisiae induction medium (SCIM) media (0.7% yeast nitrogen base without amino acids or ammonium sulfate, 0.5% yeast extract, 0.5% peptone, 0.79 g/l complete supplement mixture, 0.5% ammonium sulfate) with low glucose (0.1% w/v) at a final concentration of 6 × 10^3^ cells/ml. The 0-h, LS time point occurred after 15–16 h incubation at 30° C when these cells reach 0.5–1 × 10^7^ cells/ml. For oleate induction experiments, LS cells were harvested by 5 min of centrifugation at 3300 relative centrifugal force (RCF) to remove the low glucose media, and resuspended in an equal volume of SCIM containing 0.1% glucose (w/v), 0.15% oleate (v/v) and 0.5% Tween 40 (w/v). After addition of oleate-containing media, cells were harvested for EO time points at 0.5 h and LO time points at 5 h. All samples for analysis by microarray were harvested by centrifugation of 50 ml yeast culture at 7000 RCF for 3 min to remove liquid media, then flash-frozen for ∼30 s in liquid nitrogen and stored at −80°C for up to 2 months.

ChIP-chip samples were harvested by addition of 6 ml of 37% formaldehyde to 200 ml of yeast culture, followed by shaking at room temperature on a rotator for 1 h to fix samples. Cross-linking of samples was quenched by the addition of glycine to 125 mM and shaking for an additional 5 min. Samples were then centrifuged at 3300 RCF and washed with 40 ml Tris-buffered saline (20 mM Tris–HCl, pH 7.6, 150 mM NaCl) two times, and cell pellets were flash frozen for 30 s in liquid nitrogen and stored at −80°C.

## RESULTS

**Figure 2.**
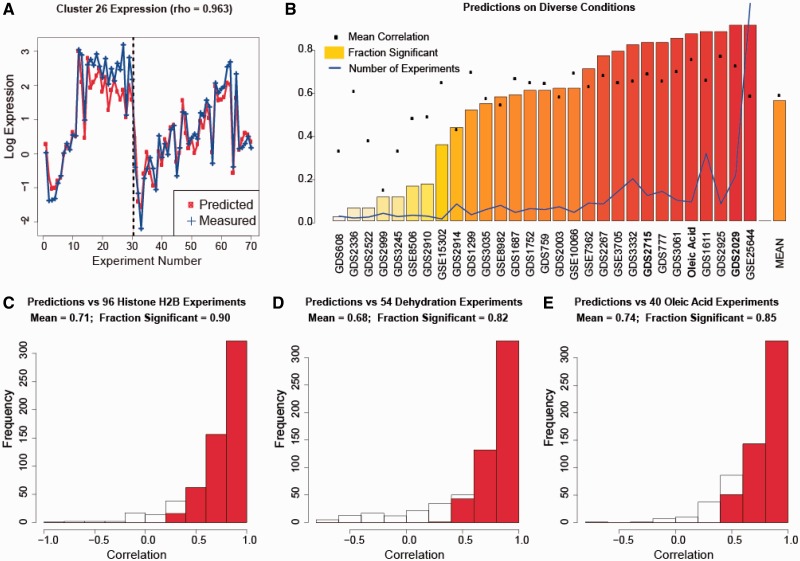
Inferelator predicts gene expression across many unseen experimental conditions. Shown are Pearson correlation coefficients between the predicted and experimentally determined mRNA expression levels for the Level 1 yeast EGRIN. The biclusters were generated using 1516 mRNA expression experiments. (**A**) The predicted and actual mRNA expression levels for a cluster in the oleic acid data set. Experiments to the left of the dotted line consist of 30 yeast-in-glucose experiments from the 1516 training examples. Experiments to the right of the dotted line consist of 40 yeast-in-oleate experiments. (**B**) The average correlation and the fraction of statistically significant correlations for 27 experiment series not present in the training set. These sets are labeled with their GEO accession numbers. The blue line labeled ‘Number of Experiments’ shows the relative fraction of experiments for each experiment set. GSE25644 has the maximum number of experiments (464), thus its relative fraction is set to 1. (**C**), (**D**), and (**E**) show the correlation for three of these experimental series: (C) GDS2029, histone H2B mutations, (D) GDS2715, dehydration and (E) oleic acid exposure (peroxisome induction). Those correlations that are statistically significant (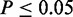) are shown in red.

This approach traverses three levels of detail as shown in Figure 1. At Level 1, a compendium of mRNA expression data (from public databases) is used to construct a global predictive EGRIN model using cMonkey for biclustering and Inferelator to infer topology of the gene regulatory network (25,26). The EGRIN model from Level 1 is predictive of cellular responses to novel environmental exposures (Figure 2) and, therefore, has captured causal mRNA expression changes within the underlying regulatory network. Importantly, interactions within this network model include indirect regulation and thus are called regulatory influences. To improve the mechanistic accuracy of the model, at Level 2, the yeast EGRIN is enhanced by combining the compendium data with a modest amount of new data that are generated under a specific and new environmental condition. Notably, these data can be relatively sparse, typical of experiments from a single laboratory and as such would, by themselves, be insufficient for construction of an EGRIN. These additional data enable the filtering of the bicluster network to include only regulatory relationships that remain coherent on exposure to the new environment and can discover new regulatory relationships not evident from the compendium data alone. That is, Level 2 analyses leverage the network structure learned from Level 1 to identify predictions from the network that are relevant to the new environmental condition and specify experiments likely to improve the network. At Level 3, approximations inherent to biclusters are shed in favor of gene by gene resolution. Regulatory mechanisms responsible for specific environmental responses are reinforced with orthogonal streams of evidence to generate a higher confidence network. Predictions from the Level 3 network enable a rational approach to designing new experiments to reveal novel biological insight and drive subsequent iterations of modeling and predictions for other environmental responses. Thus, the yeast EGRIN provides a multi-scalar picture of gene dynamics that combines predictions of gene-level and bicluster-level regulation. This framework is deliberately modular so that it is conceptually easy to add additional regulatory information, such as that from new experiments or new data types, as they become available.

We hypothesized that a network constructed from compendium data will include responses to environments not explicitly used for its construction due to linked environmental responses. For example, many conditions may be stressful to cells, and the stress response may also include anticipatory behavior that would prime the cell for peroxisomes should they be useful for dealing with a specific stressor such as oleic acid. Building from compendium data, a general yeast EGRIN model was established and exploited to rationally design experiments that inform an oleate-specific network model revealing novel mechanisms operating during the response. Oleic acid treatment tested the ability of our yeast EGRIN to uncover novel peroxisome-related regulation, although oleic acid exposure was not part of the initial training set. This test demonstrated that EGRIN predictions enabled rational selection of TFs for elucidating regulatory mechanisms by measuring the consequences of gene deletions and mapping genome-wide binding locations.

### Level 1: Globally predictive gene regulatory network

Large biological data sets tend to be noisy. Predicted regulation based on analysis of these data often report interactions that do not exist and to fail to report many interactions that do exist. However, we expect these predictions to be correct more often than incorrect, and in aggregate to provide useful information (48). Thus, we filter predictions of interactions through clusters of genes and only keep those that are reported a significant number of times. By aggregating the putative interactions, we expect to enrich the number of true regulatory events. We use this aggregation to identify candidate regulators that are likely to regulate genes of a certain type (e.g. genes in clusters enriched for peroxisome genes).

The Level 1 yeast EGRIN was constructed from a compendium of 1516 transcriptional profiles for all yeast genes (49) as shown in Figure 1A.1. These data were used to construct linear models describing which regulators control which genes (Figure 1A. 2) and augmented with protein interaction data to make condition-specific clusters of genes (i.e. biclusters). These biclusters were constructed using cMonkey (25), which generates a probabilistic framework from protein interactions and functional associations (50) and shared *cis*-regulatory promoter elements to iteratively test genes for co-expression over subsets of environmental conditions (25,45). The regulatory model was constructed using Inferelator, a regression-based approach that models the relative changes in the gene expression as a linear combination of temporally preceding changes in the activities of putative regulators (26). At this level, the EGRIN considered 406 proteins as putative regulators to build linear regression models for each of the >6000 genes in *S. cerevisiae*. The 406 proteins include 403 unique TFs, basal TFs, kinases, phosphatases and cofactors, as well as other regulators such as polymerases and histone-related proteins present in http://biochemie.web.med.uni-muenchen.de/YTFD/YTF_alpha_2.htm (51) plus three known factors not present in the database (open reading frames: YIL036w, YKR064w and YOR363c). To compensate for the noise in the data and the stochasticity in Inferelator, we identified proteins that were significantly enriched (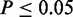) as regulators for the genes in biclusters (Figure 1A.3). We then considered only those biclusters that were significantly enriched for genes encoding peroxisomal proteins (‘peroxisome genes’) and weighted the regulators of those clusters by their *P*-values to build a prioritized list of peroxisome-regulating candidates for further study (Figure 1A.4). From this analysis, the global data set revealed Mbr1p, Cat8p, Sip4p, Adr1p and Hap4p as the top five candidates for peroxisome regulation.

We tested the EGRIN trained on the 1516 experiments (49) and using 406 possible regulators against 26 new data sets taken from the Gene Expression Omnibus (GEO) (7). These new data sets did not appear in the compendium data set, tended to have large numbers of experiments, and were not used to generate the EGRIN. We were interested in EGRIN’s ability to capture yeast responses to all conditions, and thus the new data were derived from experiments under conditions that were both similar to, and different from, the environmental conditions used to generate the compendium data (Supplementary Table S1). To determine which predictions were statistically significant, we shuffled the experiments (*n* = 1000) to calculate an expected background distribution of correlation between the predictions and the experimental results. Shown in Figure 2, are the predicted and measured expression levels within a single bicluster (Figure 2A), aggregate correlations for all data sets (Figure 2B) and a histogram showing the breakdown of correlations for 96 histone H2B mutation experiments (Figure 2C) (52,53), and 54 dehydration experiments (54) (Figure 2D). Because the EGRIN constructed from the compendium data was able to accurately reconstruct gene expression in peroxisome-proliferating oleic acid experiments (Figure 2E), we considered it sufficiently accurate to identify factors regulating genes related to peroxisomes.

As shown by the generally high proportion of significant correlations in Figure 2B, there is sufficient information within the compendium of gene expression profiles to predict condition-specific regulation even though the specific environmental conditions were not intentionally perturbed in the training set experiments. Other than conditional similarity, there are two related explanations why this is possible. First, the ‘new’ environmental conditions stimulate inextricably linked and coordinated responses. For example, oleic acid exposure is coordinated with a general stress response (19,20,29). Second, the organism may ‘anticipate’ a related response that is normally coordinated with the environmental conditions that are used as a perturbation. For example, yeast glucose metabolism typically results in ethanol production, so it is reasonable to expect that ethanol resistance is anticipated in glucose metabolism.

In either case, environmental responses appear to be tightly coupled in nature (18,55,56) and we can exploit this to build generally predictive network models from a modest number of environmental perturbation experiments. In support of this idea, the best predicted responses were associated with natural reversible physiological responses and less well for unnatural conditions—gene expression levels were predicted remarkably well for most conditions, but not for exposure to caffeine.

### Level 2: Condition-specific enhancements

Level 1 analysis enabled the development of a model learned from a compendium of transcriptome profiles. This model accurately predicted gene expression changes under novel environments that induce specific physiological responses. However, during the course of this study, we generated several yeast-in-oleate experiments including a time series from 30 min to 5 h after oleate induction. This was combined with other experiments to result in a 70-experiment oleate-specific data containing time series experiments of yeast cells exposed to oleic acid (40 experiments) and glucose (30 experiments) from different laboratories and varying conditions (20,29,57,58). Previous experiments (18,29,59) suggest at least two stages in yeast oleic acid response: one occurs between 0 and 1.5 h, and the second occurs beyond 1.5 h. Accordingly, we define EO response as the set of events that occur within 90 min in oleic acid and LO response as that which occurs after 90 min. We capture events in these two stages by harvesting cells pre-oleic acid exposure, i.e. under LS conditions at 0 min (the resting state), and at two points post-oleate exposure, i.e. EO conditions at 30 min, and LO conditions at 5 h. At Level 2, we combined this condition-specific data with the compendium data to improve the quality of the global network and the candidates for peroxisome regulation.

Condition-specific data were combined with the compendium data using the three filters shown in Figure 1B. In the first filter, we artificially reduced the compendium data set to include only those target genes and putative factors whose expression changed significantly during the yeast-in-oleate experiments (Figure 1B.1). Gene deletion studies revealed that this resulted in a five-point improvement (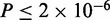) in the classifier’s ability to identify regulator targets (Table 1). We also used the analysis shown in Figure 2E to include only those predictions that were shown to be accurate when yeast was grown in oleic acid (Figure 1B.2). This resulted in a similar five-point improvement (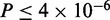) in the classifier’s ability to identify regulator targets (Table 1). Importantly, fatty acid (oleic acid) exposure, which induces peroxisomes, was not included in the compendium data set; yet, the (aggregate) correlation between EGRIN predictions of bicluster expression and measured bicluster expression was 0.74. On comparison of these predictions with the background model, 85% of the predictions were significant (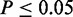). We then ran the 70-experiment yeast-in-oleate data through the procedure outlined in Level 1 and the filters outlined in Level 2. This generated clusters that were more relevant to oleate-acid response and many that were coherent under some combination of LS, EO and LO conditions (Figure 1B.3). This resulted in a three-point improvement (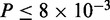) in the classifier’s ability to identify regulator targets (Table 1). Taken in aggregate, the three filters improved the classifier by 10 points (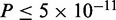), and removed two-thirds of the predictions tested by our microarray deletions (Table 1). We then ranked the remaining putative regulators of peroxisomes by their *P*-values and by how frequently the cluster they were regulating was coherent.

**Table 1.** Level two filters improve classifier accuracy

EGRIN	Agreement	Correlation	Agreement.fix	Correlation.fix	Numbers of predictions	*P*-value
Compendium	0.51	0.16	0.66	0.33	3145	1.00E + 00
Filter 1	0.51	0.18	0.71	0.44	2012	1.82E − 06
Filter 2	0.49	0.20	0.71	0.40	2086	3.09E − 06
Filter 3	0.49	0.16	0.69	0.36	1881	7.22E − 03
Aggregate	0.50	0.17	0.76	0.50	1015	4.96E − 11

‘Agreement’ and ‘Correlation’ refer to the fraction of time that Inferelator identifiers a regulator as an activator or repressor of a target gene and the target gene significantly decreases or increases in expression (respectively) when the regulator is deleted. The ‘.fix’ columns refer to the same measurements, but with the activator/repressor role of predictions swapped when they are significantly anti-correlated with the role revealed by the gene deletion assays. This swapping is intended to more accurately reflect the classifiers ability to identify peroxisome-related regulators. ‘*P*-value’ refers to the binomial *P*-value comparing the ‘Agreement.fix’ scores with those in the first column.

**Figure 3.**
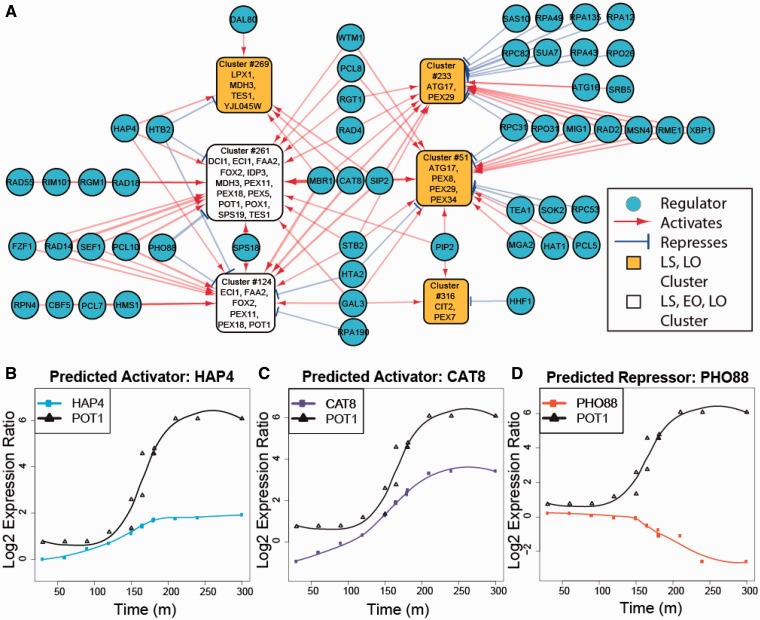
Predicted regulators of peroxisomal biclusters. (**A**) The clusters significantly enriched for peroxisome-related genes and the regulators predicted by linear regression analysis. Of the AOPY motif, only Pip2 is shown because only Pip2 mRNA levels change significantly in the yeast-in-oleate experiments. (**B–D**) Time course mRNA expression levels for pairs of regulators and their predicted targets. Lines show smoothed expression levels and symbols show raw data. Some time points (such as 180 min) have several replicates, which are visually offset by 1 minute to avoid overlap. (B) Hap4 and putative target Pot1 (a member of clusters #124 and #261) mRNA expression profiles shows a possible delayed regulation where the Hap4 increase precedes Pot1 increase. (C) Cat8 and putative target Pot1 mRNA expression profiles show a similar delayed regulation where the Cat8 increase precedes Pot1 increase. (D) Pho88 and putative target Pot1 are mRNA expression profiles anti-correlated, possibly indicating regulation that occurs at time scales finer than the sampling rate for the experiments making unclear if Pho88 expression levels precede Pot1 levels. Pho88 is not a known TF, but rather is a poorly studied putative phosphate transport protein.

For the final yeast EGRIN, we combined the regulatory program generated from the 1516 experiment compendium data set with the program from the 70-experiment condition-specific data set, giving the condition-specific program priority wherever there was a conflict. This was done so that the effect of the small number of condition-specific experiments was not suppressed by the larger data set. The outcome of this combined analysis identified a ranked list of 53 (out of an initial list of 406) putative regulators of the 6 coherent clusters enriched for peroxisome-related genes (Figure 3 and Table 2).

**Table 2.** Top predicted regulators of peroxisomes

Regulator	Total score	Type	Complex	Notes		Total score	Type	Complex	Notes
**CAT8**	173	TF			**RAD14**	77	DNA		
**MBR1**	159	TF		Suppresses Hap 2–4 deletion	**RAD2**	47	DNA		
**GAL3**	52	TF			**RAD4**	29	DNA	RAD23	
**SPS18**	52	TF?		Putative	**RAD55**	21	DNA	RAD57	
**HAP4**	48	TF	HAP 2–5		**CBF5**	12	DNA		Centromere binding factor
**FZF1**	43	TF			**RAD18**	10	DNA	RAD6	
**MIG1**	41	TF		Regulated by SNF1 and GLC7	**PHO88**	74	Other		Membrane phosphate transport
**RME1**	36	TF	IME1	Cell cycle	**MGA2**	8	Other		Membrane OLE1 transcription
**MSN4**	30	TF		Stress response	**SAS10**	8	Other		Makes 18S rRNA
**PIP2**	29	TF			**ATG16**	8	Other		Authophagy
**XBP1**	27	TF		Repressor	**HTB2**	50	Histone		
**SEF1**	26	TF		Putative	**STB2**	47	Histone	SIN3	Maybe TF or deacetylase
**WTM1**	26	TF	Ribosome		**HTA2**	22	Histone		
**RIM101**	21	TF		Repressor	**HAT1**	9	Histone	HAT2	
**RGT1**	17	TF		Activator and repressor	**HHF1**	6	Histone	HHF2	Telomeric silencing
**SOK2**	17	TF		Kinase regulator	**RPO31**	21	RNA	C160	RNA polymerase III
**HMS1**	14	TF			**RPA135**	16	RNA	A135	RNA polymerase I
**RGM1**	14	TF		Putative	**RPC31**	12	RNA	C31	RNA polymerase III
**RPN4**	12	TF		Proteasome activator	**RPA43**	10	RNA	A43	RNA polymerase I
**DAL80**	8	TF			**RPO26**	10	RNA	ABC23	RNA polymerase I,II,III
**SUA7**	8	TF		General transcription	**RPA190**	9	RNA	A190	RNA polymerase I
**TEA1**	6	TF		Ty1 enhancer activator	**SRB5**	8	RNA		RNA polymerase II
**SIP2**	161	Kinome	SNF1		**RPC82**	8	RNA	C82	RNA polymerase III
**PCL10**	79	Kinome	PHO85		**RPC53**	7	RNA	C53	RNA polymerase III
**PCL8**	24	Kinome	PHO85		**RPA12**	7	RNA	A12.2	RNA polymerase I
**PCL7**	22	Kinome	PHO85		**RPA49**	6	RNA	A49	RNA polymerase I
**PCL5**	6	Kinome	PHO85						

Type refers to the type of regulator: TF, a transcription factor; Kinome, a member of a Phophatase or Kinase complex; Histone, a histone modification protein; DNA, a DNA repair protein. Sps18 is labeled ‘TF?’ to emphasize that it is not proven to be a TF. Complex shows some known interaction partners.

Earlier studies on induction of yeast peroxisome biogenesis in response to oleic acid treatment revealed four TFs as particularly important: Adr1p, Oaf1p, Pip2p and Oaf3p (the AOPY motif) (18). These TFs strongly coregulate peroxisome formation in response to oleic acid exposure, but at least one of these proteins (Pip2p) has a delayed response; in these experiments, Pip2p was expressed at basal levels until mRNA levels increase dramatically after 2 h of oleate induction. This implies a gap in our understanding of early peroxisome proliferation in response to oleic acid, i.e. we do not know the sequence of events that precede and are responsible for the delayed induction of Pip2p.

This analysis identified biclusters that are significantly enriched for peroxisome-related genes (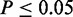) and thereby their putative Inferelator-predicted regulators in the yeast EGRIN (Figure 3). Notably, the expression profiles within most of these biclusters are not coherent under LS or EO conditions, implying that genes within these biclusters are not coregulated under glucose depletion conditions or during early stages of oleate induction. In contrast, Clusters #261 and #124, the two biclusters most heavily enriched for peroxisome annotated genes (*P* ≤ 10^−^^4^ with 16 of 29 and 8 of 20 genes, respectively), are coherent under all conditions.

The mRNA level changes of putative regulators inform regression-based inference of the regulatory network for corresponding expression changes of genes within the biclusters. The 53 putative regulators suggest one of several possible regulatory mechanisms. The regulators could be part of larger complexes, they could have competitive or cooperative functions, they could interact with ‘exclusive or’ logic, they could represent a cascade of events where some regulators act indirectly through intermediate factors (including some that are in this network diagram) or they could be downstream targets that are activated along with peroxisome-related genes. Similarly, the yeast EGRIN does not have prior knowledge of a regulator’s preferred role as activator or repressor and it may incorrectly identify a repressor as an activator (or vice versa) if the expression levels of both regulator and target are rising (or falling). Thus additional data are necessary to differentiate between these different possibilities. Nonetheless, the Level 2 network has helped to significantly constrain these follow-up studies to a manageable number of regulators (53 out of 406) in a ranked list and target genes that are specifically associated with oleate response.

As shown in Table 2: Cat8p, Mbr1p, Gal3p, Sps18p and Hap4p were the top five predicted TF regulators of clusters enriched for peroxisome-related genes. We performed gene deletion assays on the five genes encoding these proteins. Additionally, we focused on Cat8p and Hap4p for binding studies because deletion mutants, *cat8* and *hap4*, have reported defects of fatty acid utilization (19). In those experiments, in addition to fatty acid exposure, they showed defects in growth on acetate, consistent with their previously reported roles in regulating genes involved in respiration (60). Therefore we generated protein–DNA interaction maps by ChIP-Chip for both TFs (Supplementary Tables S4–S6). All experiments (interactions and expression profiles) were performed in LS, EO and LO conditions.

### Level 3: Gene level analysis

The Level 2 experimental results facilitate the transition from a bicluster-level analysis to a gene-level analysis. While the regulatory influences predicted in the yeast EGRIN are helpful, we expect some of these influences are indirect and therefore additional streams of evidence are necessary to reveal the underlying mechanisms. For example, a physical map of protein–DNA interactions would be useful to determine which of the predicted regulatory influences are due to direct binding of a TF to the promoter of regulated gene(s). At Level 3, we only consider those regulatory relationships that are reinforced by two or more streams of evidence (including expression time course data, TF deletion data, ChIP-CHIP data and TF binding motif data). Furthermore, if the co-expression of genes in a bicluster is disrupted when a putative regulator is deleted (i.e. ‘bicluster shattering’), this is evidence that the putative regulator does indeed regulate the genes in the disrupted bicluster. (See Materials and Methods for more details about these steps.)

**Figure 4.**
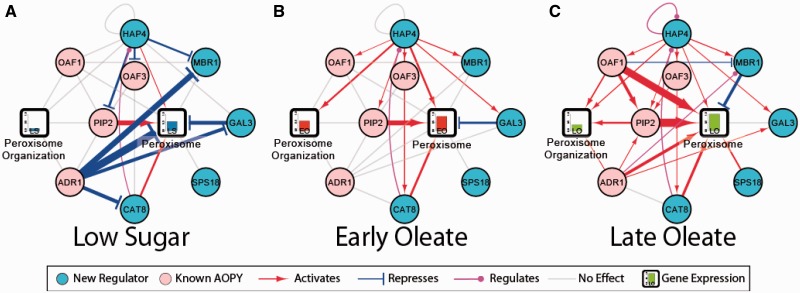
New factors interact with known AOPY to regulate peroxisomes. Regulatory influences (edges) as revealed by two or more streams of evidence in (**A**) LS, (**B**) EO and (**C**) LO. Line thickness is determined by the average –log_10_ of quantile normalized *P*-values. The bars in the peroxisome GO blocks show the normalized average expression of genes with the ‘peroxisome’ and ‘peroxisome organization’ GO annotation in the wild-type genetic background at LS, EO and LO conditions.

Figure 4 shows the five factors selected by the EGRIN and the 4 AOPY proteins with regulatory interactions that are reinforced by two or more streams of evidence. As shown, all five factors are significant regulators of peroxisome-annotated genes. Furthermore, Hap4p regulates most of the other factors as well as the downstream peroxisome genes, but is only being potentially regulated by Cat8p. Taken as a whole, this diagram suggests several regulatory circuits that would expand the core AOPY motif. See Supplementary Data S5 for the Cytoscape visualization of these data, which includes more details about the streams of evidence that informs each of the edges.

Interestingly, if we had selected only the top regulator from Table 2, Cat8p, it could have naturally led us to Mbr1p, Gal3p, and Hap4p as follows: Protein–DNA interactions of Cat8p mapped with ChIP-chip binding assays reveal a significant binding event in the promoter of HAP4. This interaction is further reinforced by the presence of a Cat8p binding motif in the promoter of HAP4. Hap4p would be predicted to regulate GAL3 due to the presence of a Hap4p binding motif in the GAL3 promoter region and a significant change in GAL3 expression in the hap4 mutant. Similarly, Hap4p would be predicted to regulate MBR1 due the shattering of a bicluster containing MBR1 in *hap4* mutants and weak evidence for a binding motif and a protein–DNA binding event. Thus, the interactions within EGRIN captured causal and biologically meaningful regulatory relationships that not only make accurate predictions of how yeast respond to new environmental perturbations but they also provide specific hypotheses regarding underlying mechanisms that can be validated by further experimentation.

### Feedback to Level 1

Guided by the yeast EGRIN, novel regulators of peroxisome-related genes were identified. These predictions were verified by targeted ChIP-chip and microarrays of selected deletion mutants. These results can then be used to iteratively improve the global EGRIN model for future analyses—for specific extension of the peroxisome-induction network, and for other gene regulatory networks of interest to the community. We therefore added the microarray time series and deletion experiments back into the compendium data set to increase the number of experiments in the training set. We tested the resulting model on 26 *S. cerevisiae* data sets taken from GEO that were not specifically related to oleate-mediated induction of peroxisomes or in the compendium data set, and this led to a significant increase in predictive accuracy [*P* ≤ 10^−^^7^, *n* = 26 (Supplementary Table S1)].

### Prediction accuracy

If EGRIN correctly predicts that a regulator is an activator or repressor of a gene, then the expression of that gene should be significantly reduced or increased (respectively) if the regulator in question is deleted. For TFs Cat8p, Hap4p, Mbr1p and Gal3p, EGRIN predictions were statistically significantly correlated (*P* 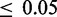) with mRNA expression profiles generated from yeast strains lacking each TF in LS, EO and LO conditions (Supplementary Table S2). We suspect that these results could be further improved if we accounted for the different metabolic states between gene deletion strains and the wild-type controls. For example, it is known that *hap4* deletion causes a decreased carbon source utilization phenotype (61,62). Thus, it may be more appropriate to compare a LO *hap4* culture with a wild-type culture consuming nutrients at a rate more similar to a LO wild-type culture. Predictions for Sps18p were less accurate than for other regulators when Sps18p influences were analysed in isolation (although they were significantly accurate when influences of other factors were also considered). This may reflect the fact that SPS18 mRNA changes were generally lower than other TFs (relative mRNA changes of SPS18 were <8% of CAT8, 4% of HAP4 and 17% of MBR1, although they were much higher than GAL3), making it more difficult for EGRIN to detect and make associated predictions.

**Figure 5.**
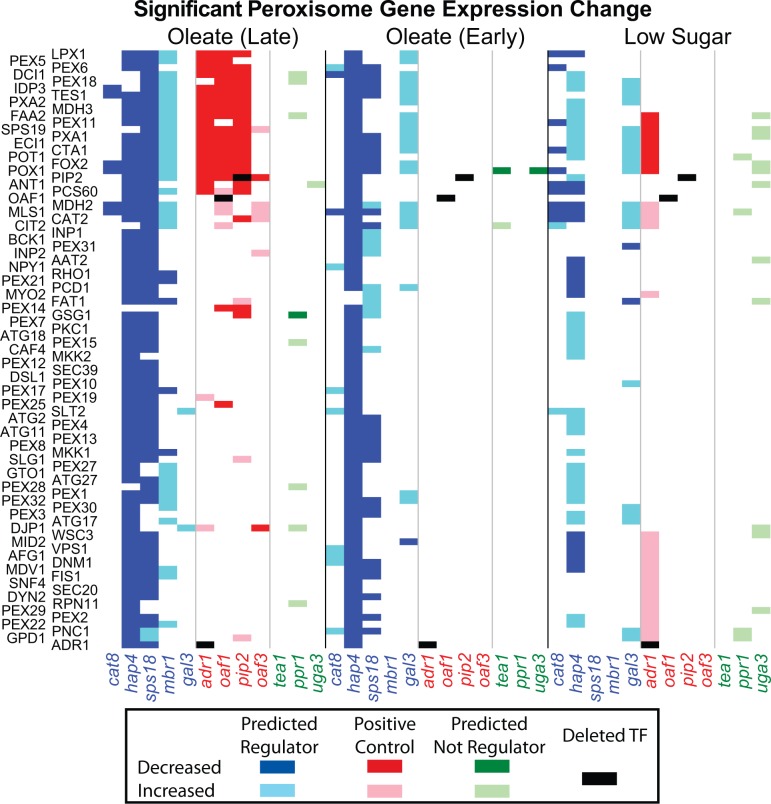
Gene deletion experiments validate model. EGRIN-predicted regulation is compared with experimentally measured significant mRNA changes (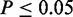) in corresponding TF deletion strains. The first five deleted genes (listed horizontally in red), the next four (in green) and the final three (in blue) are from those predicted to be regulators by the classifier, the positive control and those not predicted to be regulators by the classifier (respectively). mRNA levels were measured at 0.0 (LS), 0.5 (EO) and 5.0 (LO) h after oleate induction.

**Figure 6.**
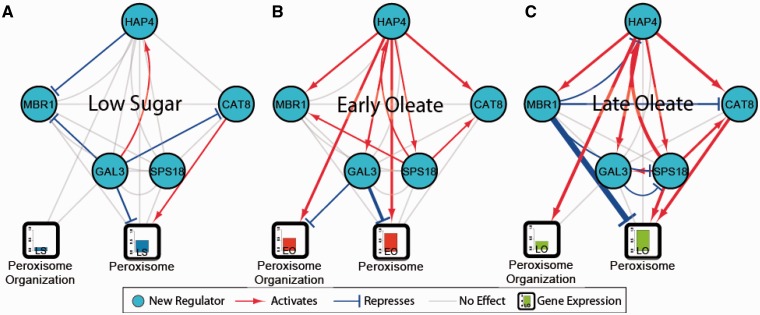
Gene deletion experiments confirm peroxisome regulation. Edges are regulatory influences revealed by microarray deletion experiments under (**A**) LS, (**B**) EO and (**C**) LO conditions. Line thickness is determined by the average 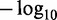 of quantile normalized 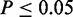. The bars in the labeled boxes show the normalized average expression of genes with the listed GO annotation in the wild-type genetic background under LS, EO and LO conditions.

To determine if the regulators selected by our method were particularly targeted toward peroxisome genes or if any stress response genes would have a similar effect on yeast in oleic acid, we chose three proteins not predicted to be regulators (Ppr1p, Tea1p and Uga3p) as negative controls. All of the genes encoding these proteins scored poorly on Table 2 (only TEA1 had any score at all) and had been identified as stress response factors (58,63). We used gene deletion studies to compare these negative controls with the top five TFs predicted to be regulators (Table 2) and a positive control, the known peroxisome regulating AOPY motif. We considered peroxisome-annotated genes that significantly changed in expression (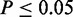) when each of the TFs was deleted. The results shown in Figure 5 reflect that the top predicted regulators significantly affect 34% of the 87 peroxisome-annotated genes across all conditions, the positive control affects 11% of the peroxisome genes, and those factors not predicted to be regulators affect 3% of the peroxisome genes (all two-tailed binomial 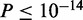). Figure 6 shows how the top five predicted regulators interact and regulate peroxisomes based on these gene deletion microarrays. See Supplementary Data S5 for an annotated Cytoscape visualization and Supplementary Data S8 for a file containing all regulatory relationships.

### Classifier stability across multiple data sets

To test different aspects of classifier stability, we trained Inferelator on 16 different data/parameter sets and cMonkey on 4 (Supplementary Data S1). By aggregating the ranked predictions across all 64 combinations, we determined that the five factors selected by our model were consistently selected across all models: Mbr1p, Cat8p, Hap4p, Gal3p and Sps18p are ranked 1st, 2nd, 4th, 6th, and 11th, respectively. These experiments also reveal the relative contributions of the compendium and oleate-specific data. In particular, the compendium data alone did not have the signals to identify Sps18p, while the Oleate-specific did not identify Hap4p. Had we combined the compendium and oleate-specific data before building the linear model, the top five factors would have been the same as the compendium data but with Pip2p (a known peroxisome regulator) substituted for Gal3p. If we had trained on all available ∼3000 publically available experiments, Ime1p and Sip4p would have been in the top five along with Cat8p, Mbr1p and Pip2p (lists of selected peroxisome regulators for different combination of data sets are included with the software package). Thus we demonstrate the overall stability of our approach regardless of minor variations in the method.

### Comparison with previous methods

Since the original cMonkey/Inferelator-based EGRIN (25,26), the gene regulatory network prediction community has tended to focus on gene-level rather than cluster-level predictions (64). To test if aggregating predicted gene regulation across biclusters improves the ability of the classifier to correctly predict gene targets, we used the gene deletion experiments to evaluate the models generated by Inferelator. We aggregated the 16 Inferelator runs through the four different cMonkey cluster sets to determine if the classifier is better at identifying genes that will significantly change in expression when the regulator is deleted, or clusters that will be enriched for genes that will significantly change in expression when the regulator is deleted. We evaluated these 16 gene level predictions and 64 cluster level predictions using the MCC, F1 and area under the ROC curve metrics (65). As shown in Table 3, the cluster-level predictions scored significantly better (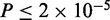) across all three metrics. Similarly, we tested to see if the aggregate predictions were better than inferred regulation based on the mean cluster signal (as was done in the original EGRIN, i.e. the Old Cluster method). We trained seven different data sets generated from combinations of the 1516 experiment compendium data set (49), the 70-experiment oleate-specific data set, and a new data set containing all experiments taken from GEO (Supplementary Table S1) on mean expression levels for clusters in the four different cMonkey cluster sets. As shown in Table 3, the aggregate cluster predictions also scored significantly better than the old style mean cluster predictions (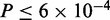) across all three metrics.

**Table 3.** Cluster level aggregation improves common machine learning metrics

Metric	Cluster	Gene	Gene *P*-values	Old cluster	Old cluster *P*-values
MCC	0.14	0.06	<3 × 10^−08^	0.06	<2 × 10^−12^
F1	0.18	0.12	<2 × 10^−05^	0.14	<6 × 10^−04^
Area under curve	0.57	0.52	<5 × 10^−08^	0.53	<2 × 10^−05^

*P*-values are calculated by using a Mann–Whitney test comparing the 16 ‘Gene’-level predictions and 28 ‘Old Cluster’-level predictions with 64 ‘Cluster’-level predictions. ‘MCC’ refers to a Matthews Correlation Coefficient, ‘F1’ to the F1 score and ‘Area under curve’ refers to the area under the receiver operating characteristic (ROC) curve.

### Comparison with other methods

Our method uses evidence of indirect regulation to identify novel regulators to test for evidence of direct regulation. For comparison, we identified two tools that combine mRNA expression data with existing interaction data to infer direct regulation without intermediate steps. Specifically, MEDUSA (9,66) and PMN (10) infer regulatory networks by correlating mRNA profiles to known or putative protein–DNA or protein–protein interactions (more details about MEDUSA and PMN are available in Supplementary Data S2 and S3). Because these algorithms were relatively slow and memory intensive, we limited the training set to the 70-experiment yeast-in-oleate data. As these algorithms build on evidence for direct regulation and work best with dramatic changes (such as from systematic deletion assays), they made few accurate predictions of TFs responsible for oleate-induced transcriptional changes. Of the five TFs highlighted in this study, MEDUSA only made predictions for Cat8p, Hap4p and Sps18p, and only those predictions for Hap4p were significantly correlated with the gene deletion experiments. Importantly, none of the five factors were predicted to regulate a statistically significant number of peroxisome annotated genes (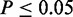). PMN did not make any predictions on the five TFs. For comparison, as shown in Supplementary Data S4, we ran Inferelator on the same data. It made significantly correlated predictions for all five factors, and predicted all but Hap4p to regulate a statistically significant number of peroxisome-annotated genes.

## DISCUSSION

Systems biology allows the construction of comprehensive, predictive regulatory networks. However, there are an infinite number of possible cellular conditions and typically large-scale gene regulatory network inference approaches fail to reveal direct, dynamic and actionable mechanisms underlying cellular regulatory responses. Here, we present a rational approach for using extant data to generate a comprehensive gene regulatory network model of relatively low granularity, which is used to guide prediction and further experimentation that iteratively improves the model. Importantly these experiments not only inform the specific mechanisms under focus, but also improve the predictive value of the global model and inform cellular responses to other perturbations. This was accomplished by building upon methods originally developed for relatively simple prokaryotes (archaea) applying them to the eukaryote, yeast.

During this study we, observed a complementarity between the regulatory network build from compendium data and the network build from condition-specific data. In particular the condition-specific data did not identify Hap4p as a regulator of peroxisomes and the compendium data did not identify Sps18p, even though both factors were later confirmed by targeted experiments. We hypothesize that Sps18p’s activity is peculiar to the oleate response and thus not included in any anticipatory responses found in the compendium data set. Conversely, we hypothesize that Hap4 is part of a more general high-level response that only becomes prominent when comparing peroxisome gene response across multiple conditions. We propose that this work combining compendium with condition-specific data establishes a foundation for researchers to, with greater predictive accuracy, explore regulatory networks specific to their areas of interest. In our specific interest, peroxisome biogenesis, the network correctly predicted several factors that influence the expression of peroxisome-related genes. It led to new players and new dynamic interactions that expand our knowledge of peroxisome-related gene induction. This analysis provides a basis for kinetic modeling and new hypotheses of the dynamic interplay controlling the cellular response. Thus we demonstrate that the regulatory hypotheses provided by a eukaryotic EGRIN can provide valuable guidance for biological experimentation and for discovering novel direct regulatory influences critical for rational intervention into cellular networks.

It is important to emphasize that there was sufficient information in the publically available experimental data set to predict condition-specific regulation under conditions not intentionally explored when that data was generated. Predictability under these conditions is likely due to coupled or anticipatory behavior of cellular regulatory networks, and this is a significant advantage for systems biology. Often condition-specific data are difficult to obtain, for example, in the case of a pathogen within its (human) host. In such a case, as we show here for the response to oleate, we would predict that compendium data would make it possible to generate a sufficiently accurate host (and pathogen) regulatory response model that would require relatively few follow-up experiments to elucidate specific mechanisms of regulation. Furthermore, the methods outlined here could, in principle, have been performed using any number of clustering and regulatory inference software packages (64,67). Thus, these methods represent an important practical advance in applying computer models to improve the pace of experimental biology.

### MBR1 and GAL3 activity reverse of predicted

As shown in Figure 5, the EGRIN predictions for Mbr1p and Gal3p were significantly anti-correlated with the behavior revealed by the *mbr1* and *gal3* microarray deletion. However, Mbr1p and Gal3p and levels are correlated with the target gene POT1 (See Supplementary Figure S1). Therefore the predictions are consistent with microarray time course data, and the target genes are well identified; however, these TFs are incorrectly identified as activators when they are really repressors. We suspect this is due to feedback inhibition in the yeast regulatory networks, and such regulation would be difficult to properly detect using the existing Inferelator framework. As ∼66% of the regulation predicted by Inferelator on our data is activation, we suspect this is a widespread problem. One solution may be use existing information about whether a TF is a known activator or repressor to improve the predictions.

### Expansion from the core regulatory network model of oleate-induced peroxisome protein expression

**Figure 7.**
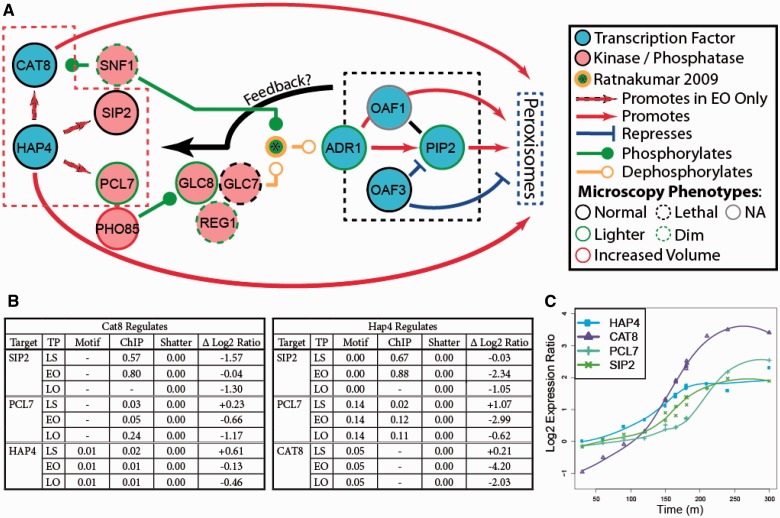
Model of possible Hap4-dependent phosphorylation in oleate. (**A**) The four regulators surrounded by the dotted red line were identified by the yeast EGRIN in Level 2 as shown in Table 2. The four regulators surrounded by the dotted black line are the well-studied AOPY peroxisome regulatory motif (18,73). The microscopy phenotypes are peroxisome growth observation taken from (17) and (75). The interactions between Snf1 and Gcl7, which promote phosphorylation of Adr1 and induction of peroxisomal genes through the AOPY motif, are discussed in (76,77) and are indicated by the starred green circle. The feedback is discussed in the text. (**B**) The evidence for the Hap4p and Cat8p regulation. Motif, ChIP-chip and Shatter show estimated *P*-values as detailed. 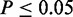 may be considered strong evidence, 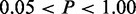 may be considered weak evidence and *P* = 1 are labeled ‘−’and are considered no evidence. The Δ Log2 Ratio is the change in log_2_ expression ratio versus wild type at the appropriate time point when *CAT8* or *HAP4* is deleted. (**C**) Shows the log_2_ expression ratios versus LS wild type in oleate induction batch culture experiments.

In *S. cerevisiae*, peroxisomes are induced in response to oleic acid, and the transcription of many peroxisomal proteins is controlled by oleate response elements, which are recognized by the fatty acid-bound heterodimer Oaf1-Pip2. This heterodimer operates within the context of a feed-forward transcriptional network involving four core TFs: Adr1p, Oaf1p, Pip2p and Oaf3p (AOPY). Constitutively expressed Oaf1p forms a heterodimer with Pip2p, which feeds back on the expression of Pip2p in an ASymmetric Self-Up-REgulating (ASSURE) motif (18,68–70). PIP2 and genes encoding many oleate-responsive peroxisomal proteins are also induced by Adr1p, thus forming a coherent type 1 (71) feed-forward loop (FFL) (18,72,73). Oaf3p drives a coherent type 2 (71) FFL modulating expression of oleate-responsive genes (18). The behavior of this network has been extensively modeled (73,74), but this network operates in the context of the larger genome. The EGRIN-based model enables expansion of the regulatory network from the core toward the entirety of the regulatory dynamics. However, as shown in Figure 4, the yeast EGRIN misidentifies Oaf3p as an activator of *PIP2*, pointing to the necessity of detailed follow-up experiments to determine specific regulatory mechanisms. Figure 7 shows a model (inspired by Table 2 and other evidence taken from the literature) ripe for such additional experimentation.

In addition to the known AOPY activity, this study revealed peroxisomal regulation by five TFs: Cat8p, Mbr1p, Gal3p, Sps18p and Hap4p. By considering the targets of these TFs that were reinforced by at least two complementary and reliable streams of evidence, we were able to determine that these TFs regulate 69 of 87 genes encoding for peroxisome or peroxisome organization–annotated proteins (Supplementary Table S8). Perhaps more interestingly, we found evidence that Hap4p regulated AOPY through a method other than just direct transcriptional regulation. Adr1p is a major component of the AOPY motif, and its activity is regulated through phosphorylation. However, the mechanism of its phosphorylation is not entirely understood (76,77). We were able to combine our EGRIN with previously unconnected phenomena appearing in the literature to shed light on the phosphorylation of Adr1p.

Thus, we propose additional activities for Hap4p. Based on our gene deletion and ChIP-chip experiments, we propose that Hap4p regulates the expression of two of the genes encoding ‘kinome’ proteins presented in Table 2: Sip2p and Pcl7p. This is particularly interesting, as these ‘kinome’ proteins are known to regulate Adr1p, as described below. As shown in Figure 7A, Sip2p is a beta subunit of the Snf1p kinase complex, a known regulator of both Cat8p and Adr1p by phosphorylation in response to glucose depletion. Cat8p is directly phosphorylated by Snf1p, but Adr1p is activated by Snf1p-dependent dephosphorylation at Ser-230 (76,77). Pcl7p is one of the subunits for the PHO85 kinase complex and one of two (along with Pcl6p) that are the usual activators of Glc8p (Pcl8p and 10 p are less frequent activators). Glc8p, along with Reg1p, is a member of the Glc7p phosphatase complex. The specific evidence for Hap4p and Cat8p regulation is shown in Figure 7B and C. The Ser-230 dephosphorylation of Adr1p (marked in the center of Figure 7A with an asterisk surrounded by a green and orange circle) is not entirely understood; however, it does involve interaction between Snf1p and the Glc7p phosphatase complex (78–80).

### Integration with additional experiments

Included in Figure 7A are phenotypes revealed by confocal fluorescence microscopy designed to assess peroxisome morphology in a battery of yeast mutants (75). Confocal fluorescence microscopy assays revealed no abnormal peroxisomal phenotype when *cat8* or *hap4* was deleted; however, these experiments examined a late time point, after a compensatory mechanism might have been engaged. Analysis of Adr1p targets revealed that Adr1p regulates PHO88, PHO85 (and PCL8), as well as CAT8, possibly pointing to a feedback mechanism whereby Adr1p in turn regulates CAT8. While abnormal peroxisomes have not been observed for *cat8*, *hap4* or *sps18* deletions, they have been for several ‘kinome’ genes in Table 2 (17,80). The two genes encoding kinases (SNF1 and PHO85) are known to have major effects on peroxisome biogenesis. Abnormal peroxisomal phenotypes are particularly pronounced in deletions of genes downstream of and including *pcl7*. This is especially significant as few related factors that were tested by confocal microscopy have phenotypes (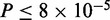).

## CONCLUSION

Systems biology promises to broaden and deepen our understanding of complex biological phenomena through the analysis of high-throughput data integrated with existing scientific knowledge. It provides models of biological activity for specialists, such as molecular biologists, to test with detailed experiments. Here we demonstrate that global compendium data compiled from public databases can be used to construct a network that correctly predicts gene expression under novel conditions by spanning the hierarchy of regulation—from signaling to transcription. The integrated network perspective provides a road map to traverse the multiple scales within this hierarchy and addresses a major challenge in systems biology that is critical to understanding regulation. The specific framework we developed for this study is modular in terms of the specific algorithms that can be deployed and extensible in terms of the data types that can be analysed. These results establish a condition-specific oleic acid network that makes numerous predictions for experimentation to further our understanding of peroxisome regulation. The globally predictive aspect of our yeast EGRIN should be helpful to anyone studying yeast regulation, but most importantly, we have demonstrated a general framework applicable to other organisms and other data types—making it possible to apply EGRINs to ever more important biomedical challenges.

## SUPPLEMENTARY DATA

Supplementary Data are available at NAR Online.

## FUNDING

National Institutes of Health [P50 GM076547, R01 GM075152, 1R01 GM077398 and U54GM103511]; National Science Foundation [DBI-0640950]; Office of Science, Office of Biological and Environmental Research, of the US Department of Energy under Contract No. [DE-AC02-05CH11231]; National Natural Science Foundation of China [31071146 and 31271365 to Y.W.]; Excellent Young Teachers Program of Southeast University [3231001201 to Y.W.] and Natural science fund in Jiangsu Province [BK2011599 to Y.W.]. Funding for open access charge: NIH [U54GM103511]; This work was partly conducted by ENIGMA (Ecosystems and Networks Integrated with Genes and Molecular Assemblies).

*Conflict of interest statement*. None declared.

## Supplementary Material

Supplementary Data
