# Supplementary material for: Molecular mechanisms of system responses to novel stimuli are predictable from public data
Source: Nucleic Acids Res. 2013 Oct 31;42(3):1442–60. doi: 10.1093/nar/gkt938 (PMC3919619; doi:10.1093/nar/gkt938)
Supplement: Supplementary Data [file supp_42_3_1442__index.html]

Molecular mechanisms of system responses to novel stimuli are predictable from public data — Molecular mechanisms of system responses to novel stimuli are predictable from public data — Supplementary Data 

# Molecular mechanisms of system responses to novel stimuli are predictable from public data

## Supplementary Data

files

**Files in this Data Supplement:**

- Supplementary Data - zip file
