# Supplementary material for: Molecular mechanisms of system responses to novel stimuli are predictable from public data
Source: Nucleic Acids Res. 2013 Oct 31;42(3):1442–60. doi: 10.1093/nar/gkt938 (PMC3919619; doi:10.1093/nar/gkt938)
Supplement: Supplementary Data [file supp_gkt938_nar-01979-n-2013-File012.zip › Supplementary Information.041113.pdf]

Supplementary Information

Table of Contents:

Supplementary Data 1: Data Sets and Aggregate Results – Page 2
Supplementary Data 2: MEDUSA – Page 3
Supplementary Data 3: PMN – Page 4
Supplementary Data 4: Inferelator – Page 5
Supplementary Table 1: Predictions on Data Sets not in Training Set – Page 6
Supplementary Table 2: Significant Changes in Peroxisome Gene Expression – Page 7
Supplementary Figure 1: Additional Gene Plots – Page 8

Supplementary Data 1: Data Sets and Aggregate Results

Over the course of these experiments we generated 8 Inferelator runs and 4 cMonkey cluster sets. For complete analysis each of inferelator was subjected to the filter shown Figure 1B.2 making a total of 16 sets of predictions. These files are released in the './PaperData' folder distributed on the cMonkey webpage and may be analyzed using the R script 'YeastOleatePipeline.R.'

These Inferelator sets are as follows:

1) Inferelator-run.OleateAndTanay.noAnd.101111.RData.hist.RData: The combined predictions used to produce Table 1 in the paper
2) Inferelator-run.tanay.noAnd.2011-10-10.97.0.1.Exp.1516.RData: The predictions trained on Tanay data considering only genes (& predictions) that pass lambda cutoff
3) Inferelator-run.tanay.noAnd.2011-10-10.98.0.1.Exp.40.RData: The predictions trained on Oleate data considering only genes (& predictions) that pass lambda cutoff
4) Inferelator-run.tanay.noAnd.2011-12-12.395.0.1.Exp.1516.RData: The predictions trained on Tanay data
5) Inferelator-run.tanay.noAnd.2012-10-30.396.0.1.Exp.70.RData: The predictions trained on Oleate data
6) Inferelator-run.tanay.noAnd.2012-10-31.392.0.1.Exp.2941.noQnormOther.RData: The predictions trained on Tanay and Other data
7) Inferelator-run.TanayOleateGlom.noAnd.2013-01-07.395.0.1.Exp.1556.RData: Trained on Tanay and Oleate data glommed together
8) Inferelator-run.TanayOleateGlom.noAnd.2013-01-08.97.0.1.Exp.1556.lamCut.RData: Trained on Tanay and Oleate data glommed together considering only genes (& predictions) that pass lambda cutoff

These cMonkey sets are as follows:

1) clusterStack.tanay.RData: The cluster built on the Tanay et al. data
2) clusterStack.tanayAndOther.RData: The clusters built on the Tanay et al. data as well as new data from SGD
3) clusterStack-2011-08-16.resplit.RData: The clusters built on the 70 experiment yeast-in-oleate data.
4) clusterStack.012413.oleate70.RData : A second set of clusters built on the 70 experiment yeast-in-oleate data.

If all 16 sets of predictions are combined with all 4 sets of biclusters, then there are 64 combinations that can be used to select top factors predicted to regulate peroxisomes. The top 25 are as shown:

Order	Factor	Score.Ave	Score.sd	Rank.Ave	Rank.sd
1	**MBR1**	107.43	66.1	2.05	1.32
2	**CAT8**	97.58	67.66	2.65	2.06
3	XBP1	36.29	33.91	7.77	8.64
4	**HAP4**	23.81	21.3	14.2	16.62
5	**GAL3**	24.32	26.5	18.64	20.96
6	**PIP2**	32.08	41.95	19.08	21.12
7	RME1	15.36	12.95	19.23	19.04
8	MSN4	10.64	10.22	23.28	20.65
9	RPN4	9.62	11.17	27.29	21.91
10	SIP4	46.85	63.47	28.89	26.42
11	**SPS18**	12.79	24.04	29.48	24.43
12	RGT1	6.97	8.11	32.52	26.02
13	RAP1	6.9	8.65	35.51	23.82
14	FZF1	11.1	15.9	36.04	26.01
15	CST6	7.24	12.9	37.18	21.28
16	IME1	16.29	28.94	37.45	24.85
17	MIG1	5.64	11.88	38.21	24.01
18	STE12	6.68	14.04	41.19	20.68
19	CUP2	4.4	8.44	41.76	20.9
20	GAT1	2.43	4.19	42.42	20.45
21	DAL80	3.97	7.85	42.75	23.09
22	HAP5	2.05	3.73	43.85	19.26
23	UGA3	1.91	3.38	44.27	19.6
24	**ADR1**	4.63	10.47	44.45	19.77
25	MAC1	2.62	5.1	44.5	18.1

Supplementary Data 2: MEDUSA

The MEDUSA algorithm (Kundaje *et al*, 2008) models the control logic of transcriptional regulation in the form of an alternating decision tree (ADT), which is a generalization of a decision tree that consists of alternating layers of decision nodes and prediction nodes. MEDUSA can integrate mRNA expression, promoter sequence and CHIP-chip occupancy data to lean a model that accurately predicts the differential expression of target genes in held-out data. For performance reasons, we used the fastMEDUSA implementation (Bozdag *et al*, 2010).

Our purpose was to estimate whether the MEDUSA built without mutation information has the capability to predict gene expression under novel perturbations. Therefore, MEDUSA was trained on a WT dataset including 40 experimental conditions for yeast in oleate and 30 experimental conditions for yeast in glucose, and generated an ADT with 2000 nodes. As MEDUSA took two days to train on this dataset, we did not attempt a larger training set. We tested the performance by traversing the resulting ADT on a deletion dataset that includes 5 deleted genes at 3 time points of 0h, 0.5h, and 5h. The results on our deleted TFs suggest that MEDUSA is unsuccessful for predicting regulators of peroxisomes.

Supplementary Table A: MEDUSA Predictions

	Agreement	Correlation	pValue	Gene Count	pValue Peroxisomes	pValue Peroxisome Organization
cat8_0.0	0.488	-0.025	0.308	254	0.08 (4/254)	NA
cat8_0.5	0.519	0.039	0.178	285	0.497 (2/285)	0.74 (1/285)
cat8_5.0	0.469	-0.081	0.107	64	0.107 (1/64)	NA
hap4_0.0	0.467	0.000	1.02E-85	722	0.521 (6/722)	0.667 (5/722)
hap4_0.5	0.948	0.000	3.55E-15	1201	0.561 (10/1201)	0.287 (12/1201)
hap4_5.0	0.993	0.000	0.003	1267	0.568 (11/1267)	0.415 (12/1267)
sps18_0.0	NA	0.000	1	0	NA	NA
sps18_0.5	0.498	0.004	0.429	896	0.063 (12/896)	0.578 (7/896)
sps18_5.0	0.309	-0.003	0.428	1767	0.14 (20/1767)	0.484 (16/1767)
mbr1_0.0	NA	0.000	1	0	NA	NA
mbr1_0.5	NA	0.000	1	0	NA	NA
mbr1_5.0	NA	0.000	1	0	NA	NA
gal3_0.0	NA	0.000	1	0	NA	NA
gal3_0.5	NA	0.000	1	0	NA	NA
gal3_5.0	NA	0.000	1	0	NA	NA
**Summary**	0.624	0.071	0	6456	NA	NA
**Fixed Summary**	0.624	0.071	0	6456	NA	NA

Supplementary Data 3: PMN

The physical module networks (PMN) is a probabilistic graphical method to learn transcriptional networks, consisting of two components: 1) Bayesian model describe modules of co-expressed genes and its shared regulation programs and 2) a Physical Interaction Graph provides possible physical interactions between proteins and genes, including protein-protein interactions, protein-DNA interactions and transcription interactions (Novershtern *et al*, 2011).

Our purpose was to estimate whether the PMN, which is built without mutation information, has the capability to predict the gene expression under novel perturbations. PMN was trained on WT dataset including 40 experimental conditions for yeast in oleate and 30 experimental conditions for yeast in glucose, and then tested on a deletion dataset of the five deleted TFs at 3 time points of 0h, 0.5h, and 5h. As PMN took three weeks to train on this dataset, we did not attempt a larger training set. The protein-protein interaction (PPI) lists for yeast are taken from String database and then filtered with score 0.01, resulting in about 7900 PPIs over 1954 proteins. The protein-DNA interaction (PDANI) lists were taken from the Young Chip dataset (Lee *et al*, 2002) and then filtered with p-value 0.001, resulting in about 6000 PDANIs over 2420 proteins and 6 deleted TFs. Note: Though we were able to get PMN to return predictions on other data sets, it did not for our data set.

The module network in PMN is a Bayesian network, which can be imprecise under sparse data conditions, such as the few deletion conditions studied herein. In addition to, the Bayesian-based methods typically involve high-dimensional integrals, which is a time/memory consuming problem. The results on our deleted TFs suggest that PMN is unsuccessful for predicting gene expression under the deletion conditions.

Supplementary Table B: PMN Predictions

	Agreement	Correlations	pValue	Gene Count	pValue Peroxisomes	pValue Peroxisome Organization
***cat8*_0.0**	NaN	NA	1	0	NA	NA
***cat8*_0.5**	NaN	NA	1	0	NA	NA
***cat8*_5.0**	NaN	NA	1	0	NA	NA
***hap4*_0.0**	NaN	NA	1	0	NA	NA
***hap4*_0.5**	NaN	NA	1	0	NA	NA
***hap4*_5.0**	NaN	NA	1	0	NA	NA
***sps18*_0.0**	NaN	NA	1	0	NA	NA
***sps18*_0.5**	NaN	NA	1	0	NA	NA
***sps18*_5.0**	NaN	NA	1	0	NA	NA
mbr1_0.0	NaN	NA	1	0	NA	NA
***mbr1*_0.5**	NaN	NA	1	0	NA	NA
***mbr1*_5.0**	NaN	NA	1	0	NA	NA
gal3_0.0	NaN	NA	1	0	NA	NA
***gal3*_0.5**	NaN	NA	1	0	NA	NA
***gal3*_5.0**	NaN	NA	1	0	NA	NA

Supplementary Data 4: Inferelator to Compare with PMN and MEDUSA

To compare with MEDUSA and PMN, we subjected Inferelator predictions to exactly the same analysis presented in Supplementary Data 1 & 2. Inferelator was trained on WT dataset including 40 experimental conditions for yeast in oleate and 30 experimental conditions for yeast in glucose, and then tested on a deletion dataset of the five deleted TFs at 3 time points of 0h, 0.5h, and 5h.

Supplementary Table C: Inferelator Predictions

	Agreement	Correlation	pValue	Gene Count	pValue Peroxisomes	pValue Peroxisome Organization
cat8_0.0	0.609	0.163	0.101	23	0.014 (1/23)	NA
cat8_0.5	0.783	0.580	0	23	0.014 (1/23)	NA
cat8_5.0	0.769	0.000	0.149	13	0 (3/13)	NA
hap4_0.0	0.526	-0.119	0.059	135	0.133 (2/135)	NA
hap4_0.5	0.925	0.797	0	159	0.185 (2/159)	NA
hap4_5.0	0.673	0.094	0.038	171	0.209 (2/171)	NA
sps18_0.0	NA	0.000	1	0	NA	NA
sps18_0.5	0.583	0.105	0.164	36	0 (5/36)	0.004 (2/36)
sps18_5.0	0.610	0.159	0	59	0 (11/59)	0 (6/59)
mbr1_0.0	0.500	0.000	0.346	4	NA	NA
mbr1_0.5	0.700	-0.167	0	10	NA	NA
mbr1_5.0	0.120	-0.677	0	250	0.169 (3/250)	0.631 (1/250)
gal3_0.0	0.092	-0.799	0	65	0.002 (3/65)	NA
gal3_0.5	0.115	0.000	1.77E-06	26	0 (5/26)	0.002 (2/26)
gal3_5.0	0.600	0.167	0.099	5	NA	NA
**Summary**	0.493	-0.124	0	979	NA	NA
**Fixed Summary**	0.737	0.467	0	979	NA	NA

Supplementary Table 1: Predictions on Data Sets not in Training Set

Supplementary Table 1: Compendium Data Set Predictions on Various Data Sets With and Without the Oleate Data. The data sets have the following GEO accession numbers: GDS1299 (Caba *et al*, 2005, caba), GDS1611 (Guan *et al*, 2006), GDS1687 (Sheehan *et al*, 2007), GDS1752 (Ronen & Botstein, 2006), GDS2003 (Lai *et al*, 2005), GDS2029 (Parra *et al*, 2006; Nag *et al*, 2010), GDS2267 (Tu *et al*, 2005), GDS2336 (Reinke *et al*, 2006), GDS2522 (Angell *et al*, 2006), GDS2715 (Singh *et al*, 2005), GDS2914 (Kuranda *et al*, 2006), GDS2925 (Abbott *et al*, 2007), GDS2999, GDS3035, GDS3061 (Komili *et al*, 2007), GDS3245 (Pan *et al*, 2008), GDS3332 (Marks *et al*, 2008), GDS608 (Prinz *et al*, 2004), GDS759 (Sapra *et al*, 2004), GDS777 (Knijnenburg *et al*, 2007; Tai *et al*, 2005; Boer *et al*, 2003), GSE15302 (Veatch *et al*, 2009), GSE25644 (van Wageningen *et al*, 2010), GSE3705 (Lai *et al*, 2008), GSE7362 (Joseph-Strauss *et al*, 2007), GSE8506, and GSE8982. P-values calculated using a two-tailed paired Wilcoxon signed-rank test. Correlations differ from Figure 2 because they were calculated on raw (rather than binary) expression values.

Experiment	Original Mean	Original Fraction Significant	w'Oleate Mean	w'Oleate Fraction Significant	Number Experiments
**GDS1299.Cytotoxic**	0.521	0.33	0.548	0.35	13
**GDS2925.OrganicAcid**	0.511	0.55	0.520	0.56	36
**GDS777.LimitedMedia**	0.505	0.66	0.514	0.67	63
**GSE3705.Antimycin**	0.498	0.58	0.512	0.6	63
**GDS2029.HistoneH2B**	0.490	0.7	0.495	0.71	96
**GDS2267.Metabolic2**	0.482	0.51	0.507	0.54	36
**GDS3061.Ribosomes**	0.480	0.55	0.484	0.54	44
**GDS1687.Microgravity**	0.475	0.35	0.478	0.36	18
**GDS3332.Wine**	0.474	0.66	0.481	0.67	90
**GDS1611.UPF1**	0.464	0.72	0.479	0.73	144
**GDS3035.Oxidative**	0.442	0.35	0.467	0.37	24
**GDS2715.Dehydration**	0.438	0.58	0.452	0.59	54
**GSE10066.Lactate**	0.429	0.33	0.442	0.33	18
**GSE15302.rho0**	0.426	0.24	0.437	0.24	4
**GDS759.SplicingMutants**	0.426	0.31	0.424	0.31	24
**GSE7362.Sporulation2**	0.424	0.49	0.435	0.5	38
**GDS2003.Anaerobic**	0.423	0.36	0.441	0.38	30
**GDS1752.CarbonSource**	0.419	0.29	0.425	0.29	26
**GSE25644.Kinome**	0.408	0.77	0.416	0.78	464
**GSE8982.Alpha**	0.375	0.31	0.376	0.32	33
**GSE8506.Sporulation**	0.340	0.11	0.344	0.11	12
**GDS2336.CaffeineTOR1**	0.330	0.08	0.334	0.08	6
**GDS2910.Stress**	0.298	0.1	0.297	0.1	10
**GDS3245.Pterostilbene**	0.246	0.11	0.267	0.12	9
**GDS2522.Pyocyanin**	0.236	0.09	0.247	0.09	8
**GDS2914.Caffeine**	0.208	0.2	0.212	0.2	36
**GDS608.Filamentous**	0.167	0.07	0.176	0.07	10
**GDS2999.Gentamicin**	0.132	0.12	0.145	0.12	16
**Oleate**	0.549	0.6	0.720	0.86	40
**MEAN (No Oleate)**	0.395	0.376	0.405	0.383	-
**p.value (No Oleate)**	-	-	**7.45E-08**	**1.00E-03**	-
**MEAN (w'Oleate)**	0.401	0.383	0.416	0.400	-
**p.value (w'Oleate)**	-	-	**3.73E-08**	**4.81E-04**	-

Supplementary Table 2: Significant Changes in Peroxisome Gene Expression

The horizontal axis shows genes deleted and the vertical axis shows peroxisome annotated genes that change significantly in expression when those genes are deleted (p-value $\leq$ 0.05). The three experimental conditions (LO, EO, LS) are separated by vertical black lines and these conditions are subdivided by grey lines into factors Selected by the EGRIN, known Positive regulators of peroxisomes, and factors Not Selected by the EGRIN. Binomial tests show that there is a statistical difference between the number of significantly changing genes as compared to the Not Selected set (p-value $\leq 10^{-101}$ for Selected, p-value $\leq 10^{-14}$ for Positives).

	Agreement	Correlation	p-Value	Program Agreement	Program Correlation	Program p-Value	# of Genes
cat8 (LS)	0.68	0.26	0.02	0.67	0.22	0.03	82
cat8 (EO)	0.78	0.58	0.00	0.81	0.64	0.00	37
cat8 (LO)	0.93	0.00	0.35	0.93	0.00	0.35	30
hap4 (LS)	0.54	0.04	0.30	0.71	0.41	0.00	190
hap4 (EO)	0.82	0.53	0.00	0.93	0.79	0.00	164
hap4 (LO)	0.64	0.02	0.48	0.85	0.13	0.07	163
sps18 (LS)	NA	0.00	1.00	NA	0.00	1.00	0
sps18 (EO)	0.48	-0.07	0.25	0.89	0.76	0.00	73
sps18 (LO)	0.64	0.04	0.21	0.77	0.26	0.00	91
mbr1 (LS)	1.00	0.00	NA	1.00	0.00	NA	5
mbr1 (EO)	0.89	0.00	1.00	0.89	0.00	1.00	9
mbr1 (LO)	0.05	-0.89	0.00	0.96	0.91	0.00	311
gal3 (LS)	0.27	-0.33	0.00	0.65	0.39	0.00	113
gal3 (EO)	0.10	-0.61	0.00	0.10	-0.61	0.00	41
gal3 (LO)	0.60	0.17	0.20	0.60	0.17	0.20	5
pip2 (LS)	1.00	0.00	NA	1.00	0.00	NA	1
pip2 (EO)	0.00	0.00	1.00	0.00	0.00	1.00	1
pip2 (LO)	0.83	0.63	0.05	0.83	0.63	0.06	6
tea1 (LS)	0.50	-0.05	0.37	0.50	-0.05	0.36	16
tea1 (EO)	0.17	-0.71	0.01	0.83	0.71	0.01	6
tea1 (LO)	0.86	0.53	0.01	0.86	0.53	0.01	14
ppr1 (LS)	0.82	0.60	0.00	0.93	0.84	0.00	28
ppr1 (EO)	0.50	0.00	1.00	0.50	0.00	1.00	4
ppr1 (LO)	0.83	0.63	0.00	0.83	0.63	0.00	23
uga3 (LS)	0.60	0.08	0.33	0.60	0.08	0.30	35
uga3 (EO)	0.33	-0.25	0.23	0.67	0.25	0.24	6
uga3 (LO)	0.67	0.19	0.29	0.78	0.36	0.20	9
**Summary**	0.49	-0.12	0.00	0.80	0.57	0.00	1463
**Fixed Summary**	0.74	0.47	0.00	0.80	0.57	0.00	1463

Supplementary Figure 1: Additional Gene Plots

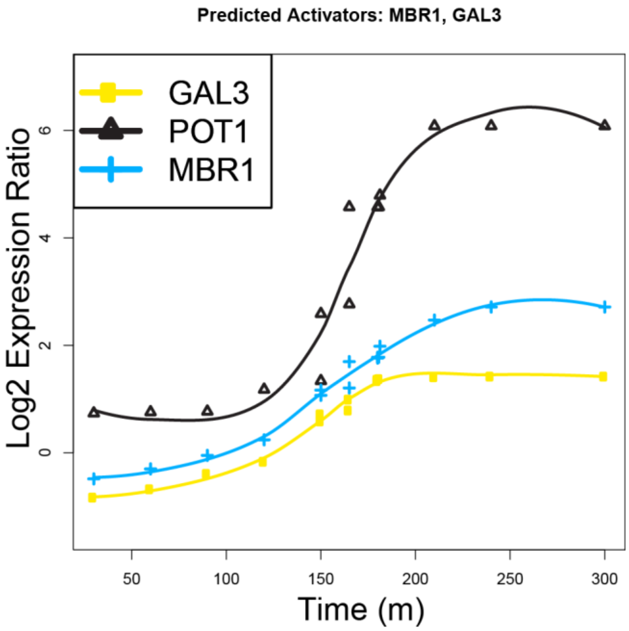

Supplementary Figure: Gal3 and Mbr1 mRNA Expression Levels
